# Multi-UAV Cooperative Coverage Search for Various Regions Based on Differential Evolution Algorithm

**DOI:** 10.3390/biomimetics9070384

**Published:** 2024-06-25

**Authors:** Hui Zeng, Lei Tong, Xuewen Xia

**Affiliations:** 1 Xinjiang Institute of Engineering, College of Information Engineering, Urumqi 830091, China; huizeng9999@163.com; 2 Hubei SME Mathematical Intellectualization Innovation Development Research Center, Wuhan Business University, Wuhan 432000, China; tonglei20200123@163.com; 3 College of Physics and Information Engineering, Minnan Normal University, Zhangzhou 363000, China

**Keywords:** multi-UAV, coverage path planning, differential evolution, dynamic reward function

## Abstract

In recent years, remotely controlling an unmanned aerial vehicle (UAV) to perform coverage search missions has become increasingly popular due to the advantages of the UAV, such as small size, high maneuverability, and low cost. However, due to the distance limitations of the remote control and endurance of a UAV, a single UAV cannot effectively perform a search mission in various and complex regions. Thus, using a group of UAVs to deal with coverage search missions has become a research hotspot in the last decade. In this paper, a differential evolution (DE)-based multi-UAV cooperative coverage algorithm is proposed to deal with the coverage tasks in different regions. In the proposed algorithm, named DECSMU, the entire coverage process is divided into many coverage stages. Before each coverage stage, every UAV automatically plans its flight path based on DE. To obtain a promising flight trajectory for a UAV, a dynamic reward function is designed to evaluate the quality of the planned path in terms of the coverage rate and the energy consumption of the UAV. In each coverage stage, an information interaction between different UAVs is carried out through a communication network, and a distributed model predictive control is used to realize the collaborative coverage of multiple UAVs. The experimental results show that the strategy can achieve high coverage and a low energy consumption index under the constraints of collision avoidance. The favorable performance in DECSMU on different regions also demonstrate that it has outstanding stability and generality.

## 1. Introduction

Coverage path planning (CPP) tasks [1] are playing an increasingly important role in many real applications, such as surveillance, surveying, and rescue. Generally, the CPP tasks are mainly divided into two categories, i.e., (1) searching for a specific target from a target region and (2) covering a target region as much as possible with (or without) prior knowledge. In recent years, the use of unmanned aerial vehicles (UAVs) to address the CPP tasks has become very popular since the UAV is a flexible and lightweight aircraft that does not require a pilot. Furthermore, compared to a manned aircraft, a UAV has higher safety and fault tolerance when performing dangerous tasks. Therefore, recently, more scholars have been investigating the use of UAVs to solve CPP tasks [2,3,4].

When using multiple UAVs to address CPP tasks, the main goal is to plan efficient coverage paths for UAVs [4,5]. Each path should enable a UAV to avoid obstacles and maintain a safe distance from other UAVs. Meanwhile, each UAV’s trajectory should satisfy some dynamic constraints [4,6,7], such as the deflection angle, maximum load, hoverability, and so on.

In the last few decades, the zigzag pattern [8] and spiral pattern [9] have become popular methods for planning coverage paths for UAVs or robots in regular or convex regions. These methods have the advantages of fewer calculations, strong fusibility, and low energy consumption. In real-life scenarios, however, most coverage search tasks are non-convex, and some tasks are even discrete. Thus, in various complicated regions, the traditional zigzag pattern and spiral pattern are no longer feasible or effective. Although some area decomposition strategies [10,11] can enhance the performance of the zigzag pattern and spiral pattern on CPP tasks to a certain extent, they have shortcomings in universality. Moreover, the strategies are unable to deal with the damage to UAVs, which is a common problem in a multi-UAV system.

In real applications, when planning an efficient search (or coverage) path for a UAV, it is necessary to use some way to represent the state of the UAV and its environment. For example, a structure called configuration space (C-space) was introduced in [4], in which information about the position and direction of each unmanned ground vehicles existed in the form of a point in the C-space at each time point. In addition, physical obstacles can also be mapped into the C-space. Thus, based on the C-space, a complicated motion problem can be converted into a point-to-point motion planning problem. In contrast, Zhang [7] broke down the search problem into three sub-problems, i.e., information fusion, task assignment, and multi-UAV behavior decision-making. Accordingly, the cooperation process among the sub-problems was conducted in a dynamic environment in order to enhance the cooperative behavior of multiple UAVs.

Considering that evolutionary algorithms (EAs) have been shown to exhibit high reliability and an outstanding performance in many scientific research areas and engineering applications, many researchers are investigating the capacity of EAs to solve the UAV path planning problems [12]. For instance, Ref. [13] designed a breeder genetic algorithm for a terrain with known final destinations. In [14], a genetic algorithm (GA) was used to optimize the path planning results and to improve the search performance of a UAV. To overcome the shortcomings of traditional EAs, the authors of [15] proposed a multiple swarm fruit fly optimization algorithm, in which several populations evolve simultaneously and compete with each other. Based on the parallel evolution mechanism, the algorithm enhances the exploration ability of a UAV and significantly reduces the probability of the planning being classified as a local optimal solution.

Although the abovementioned studies manifest that utilizing a UAV to deal with a CPP task is a promising method, a single UAV can not fulfill a complicated CPP task efficiently due to the limited endurance capability of the UAV as a result of its limited battery life [16]. Thus, using multiple UAVs to solve the CPP tasks has become a research hotspot in recent years [2,4,17]. Fortunately, the popularity of the 5G network as well as the improvement in UAVs’ performance in recent years have made it possible to conduct research on CPP tasks based on the multi-UAV cooperation mechanism. The core idea of the mechanism is to realize the cooperation of search process through information interaction between multiple UAVs so as to realize the intelligent emergence of a large-scale unmanned system [18].

In the cooperative control of large-scale unmanned systems, a primary problem is how to control the emergence of swarm intelligence. Generally, the problem can be divided into three categories [19]: (1) what kind of group behavior will the whole system produce after making local rules for individuals?; (2) what are the individual local rules for a given group behavior?; and (3) how can group behavior be controlled based on the given individual local rules? Generally, the relationship between local rules and group behavior is interactive. Thus, the design of proper local rules and control strategies is crucial for generating desired cluster system behaviors.

In recent years, there has been some research on the local rules of the unmanned system. In 2001, Desai et al. [20] studied the frame transition behavior of mobile robot formation when encountering obstacles. In the study, each robot had its own controllers and sensors, and then various local rules were formulated for different robots to realize obstacle climbing, which can be regarded as a kind of distributed control. Furthermore, Couzin et al. [21] studied a self-organization model, known as the R-A model, in a three-dimensional space. In the model, each individual has its own local rule. Concretely, each individual takes its position as the center to establish three circles with different radii: the smallest circle is called the zone of repulsion (ZoR), the largest circle is called the zone of attraction (ZoA), and the middle circle is called the zone of orientation (ZoO). Based on the three circles, each individual has three distinct movement rules to keep itself in the ZoA during cooperative movement. In 2015, Ji [22] presented a distributed collaborative search algorithm for multi-UAV clusters with limited perception and communication capabilities in a non-convex environment. An adaptive density function was designed based on a real-time updated probability map and an uncertainty map. Relying on the density function, each UAV can adjust its search direction during the search process, and the probability maps of two UAVs can be fused if the two UAVs are in a predefined communication range.

From the above research, it can be concluded that under the premise of good local rules, using a distributed system is a reasonable cluster collaboration strategy. In fact, although a centralized framework can obtain the global optimal solution for some issues [23,24,25], the increasing complexity of a problem and a large number of UAVs may cause the load of the computing center to increase significantly. As a result, the search performance of the centralized framework will deteriorate rapidly. Hence, utilizing a distributed collaborative framework, in which the total computing load is allocated on each unit, is a promising choice to deal with large-scale CPP problems.

In most studies of online path planning for UAVs, the goal is to find one (or more) optimal solution. However, it is very hard to obtain the optimal solution due to the fact that the environmental information in a dynamic and unknown environment is often incomplete. Moreover, even if the true optimal solution can be obtained, the solution may be unsuitable in many real applications. For instance, for some online path planning problems, the real-time ability is more important than an optimal route. Thus, the improvement of the real-time ability has become a research focus for such problems [26,27].

The differential evolution (DE) algorithm is an outstanding heuristic optimization algorithm, and some favorable characteristics of DE, such as a low number of parameters, easy implementation, and parallelism, have enabled it to be successfully applied in many fields. Some studies have shown that DE can offer more promising results than other evolutionary algorithms, such the particle swarm optimization algorithm and genetic algorithm. Thus, in this study, a DE-based cooperative search strategy of multiple UAVs, named DECSMU, is proposed. Its main contributions are detailed as follows:

A Distributed Model Predictive Control (DMPC) strategy is used to complete the cooperative behavior among multiple UAVs. Furthermore, the complexity of the problem is reduced by decomposing a problem into multiple time slices so as to establish the basis of UAV real-time performance.

For each UAV in a single time slice, a simplified DE is used for online path planning.

According to characteristics of different types of regions, a dynamic reward function, which can be changed with the state of the UAV, is designed to enhance the coverage ability and energy saving ability of UAVs.

The remainder of this paper is organized as follows. Section 2 introduces the problem of this paper, give the methods and conditions of map update and fusion. In Section 3, a simplified differential planning algorithm is explained, and the flowchart of DMPC is given. Section 4 introduces the dynamic reward function and gives the pseudocode of DECSMU. In addition, three sets of experiments are designed to verify the proposed method, and the simulation results, along with the analysis, are shown in Section 5. Finally, the conclusions are presented in Section 6.

## 2. Modeling of the Swarm of UAVs

In this study, each UAV is equipped with a Field of View (FOV) sensor. Multiple UAVs in a swarm are required to fly on the same plane to ensure that the proportions of the images are uniform. Moreover, two UAVs in the swarm can exchange their information when they are within a communication range, but they cannot transfer the information. The swarm of UAVs adopts a distributed collaborative search framework, in which each UAV formulates its search path based on its local information and other UAVs’ strategy information within the communication range. If necessary, the search path can be adjusted.

It is worth noting that, in real environments, an island diagram may be non-convex and discrete. Thus, when utilizing UAVs to cover and search an island terrain, the UAVs need to have the ability to search across the non-search area. Although there are some non-mission areas within an island terrain, the UAVs can fly across the non-mission areas to cover and search the island terrain more effectively.

### 2.1. Model of Mission Area

**Figure 1:**
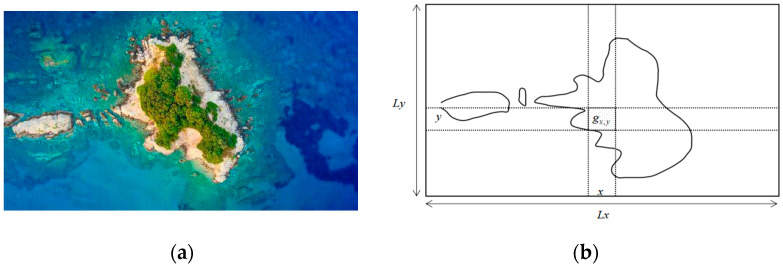
A simplified rasterized map for an island map. (**a**) A real island map. (**b**) A grid map of the island.

In order to facilitate the design and simulation of the search algorithm, this study uses a grid-based method to simplify satellite photographs of the islands. For example, the real island map demonstrated in Figure 1a can be rasterized as a simplified map shown in Figure 1b.

In Figure 1b, the task area *M* is placed in an *L_x_* × *L_y_* sized raster, where the position of any grid *g* can be represented as Equation (1).

(1) $$g_{x,y}=\left\{\left(x,y\right)\left|\left(x,y\right)\in M\right.\right\}$$

In general, this study uses discretized raster location coordinates to represent geographical locations; for example, (*x*, *y*) in Equation (1) means that the grid *g* is located in the row *x* and the column *y*. The search state for raster *g_x,y_* at time *t* can be expressed as Equation (2).

(2) $$S(g_{x,y},t)=\left\{\begin{array}{c} 0,\;\;\;g_{x,y}\in M_{n}\\ 1,\;\;\;g_{x,y}\in M_{s}\\ -1,\;\;\;g_{x,y}\in M_{u} \end{array}\right.$$

where *M_n_*, *M_s_*, and *M_u_* denote the non-mission area, searched area, and unsearched area, respectively.

### 2.2. Configuration Transfer Model

In this study, the C-space idea proposed by [4] is used to configure the state of a UAV. Suppose *N* is the number of UAVs in a swarm, *UAV_i_*, *i* ∈ [1, *NU*] stands for the *i*-th UAV, and the configuration of the *i*-th UAV at time t can be expressed as Equation (3).

(3) $$UAV_{i}=\left\{x(t),\;\;y(t),\;\theta (t)|\left(x(t),\;\;y(t)\right)\in M,\;\theta (t)\in \left[0,\;2\pi \right]\right\}$$

where *x*(*t*) and *y*(*t*) are the horizontal and vertical coordinates of the *UAV_i_* at time *t* and *θ*(*t*) is the deflection angle of the *UAV_i_* at time *t*. Thus, the configuration transfer equation of the *UAV_i_* can be obtained as Equation (4).

(4) $$\left\{\begin{array}{l} x(t+1)=x(t)+\Delta t\cdot V\cdot \cos\theta (t)\\ y(t+1)=y(t)+\Delta t\cdot V\cdot \sin\theta (t)\\ \theta (t+1)=\theta (t)+\Delta u(t) \end{array}\right.$$

where ∆*t* is a unit time slice, *V* is the flight velocity of the *UAV_i_*, and ∆*u* is the change value of deflection angle.

**Figure 2:**
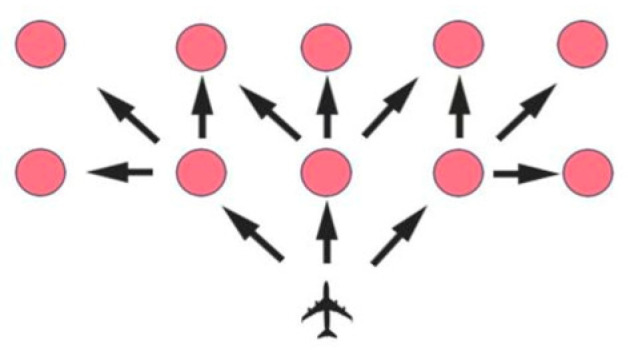
Candidate flight directions of a non-hoverable UAV in a grid map.

According to this study, UAVs can be separated into two types, i.e., hoverable and non-hoverable UAVs. When a hoverable UAV flies within a grid map, it has eight candidate search directions at each time point, i.e., front, back, left, right, left-front, right-front, left-rear, and right-rear. On the contrary, a non-hoverable UAV only has three candidate search directions at each time point, i.e., front, left-front, and right-front of the current flight directions. In this study, non-hoverable UAVs are considered, and the motion model of each UAV is shown in Figure 2, in which each red circle means the new position of the UAV and each black arrow denotes the UAV’s fly directions.

**Figure 3:**
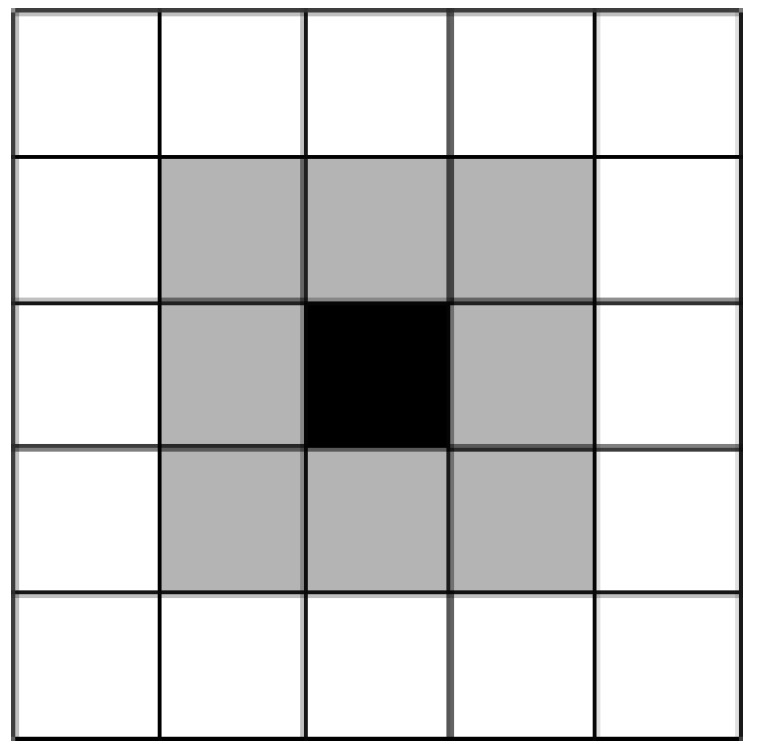
View window (view radius *r* = 1) of the *UAV_i_* in a grid map. In the grid map, the dark grid denotes the UAV’s current position, and the gray grids denote the regions that have been searched by the UAV.

Generally, a UAV has a view window during the coverage search process. Concretely, based on the grid diagram, the view window of *UAV_i_* can be represented by a view radius *r*. For example, at time *t*, if the position of *UAV_i_* is (*x*(*t*) and *y*(*t*)); then, the view window *W_i_*(*t*) of the *UAV_i_* can be represented as (*x*(*t*) ± *r* and *y*(*t*) ± *r*). In this case, we can assume that the regions within the view window have been searched. The view window of the *UAV_i_* at time *t*, denoted as *W_i_*(*t*), in a grip map is shown in Figure 3.

### 2.3. Map Update and Integration

When using multiple UAVs to perform a coverage search task, each UAV needs to know the current coverage state according to its own local map. If two UAVs are within their communication range, they can share their maps. In other words, two local maps need to be integrated into a new map, which can be shared by the two UAVs.

For example, the local map of *UAV_i_* is *M_i_*, which is initialized as *M_i_* = *M*. Then, the local map status at time *t* is represented by *S_i_*(*g_x_*_,*y*_, *t*). Based on the search window, *UAV_i_* can move to the next position and then update its local map information. The update rule of the local map information can be defined as Equation (5).

(5) $$S_{i}(g_{x,y},t)=\left\{\begin{array}{c} 1,\;\;\;\;\mathrm{if}\;g_{x,y}\in W_{i}(t)\;\mathrm{and}\;S_{i}(g_{x,y},t-1)\neq 0\\ 0,\;\;\;\;\mathrm{if}\;g_{x,y}\in W_{i}(t)\;\mathrm{and}\;S_{i}(g_{x,y},t-1)=0 \end{array}\right.$$

During the search process, if two UAVs within a same communication range and the local maps of the two UAVs can be integrated, then, more complete environmental information is obtained. Because the communication network changes dynamically with time, we need to use a dynamic communication topology matrix to represent this relationship. In this study, the communication condition at *t* is defined as Equation (6).

(6) $$c_{ij}=\left\{\begin{array}{l} 0,\;\;\;dist_{x,y}\left(t\right)>cd\\ 1,\;\;\;sd<dist_{x,y}\left(t\right)\leq cd \end{array}\right.$$

where *c_ij_* is the communication condition between *UAV_i_* and *UAV_j_*; *dist_x_*_,*y*_(*t*) represents the distance between *UAV_i_* and *UAV_j_* at *t*; *cd* represents the communication distance; and *sd* represents the safety distance.

When *c_ij_* = 1, the *UAV_i_* and *UAV_j_* can communicate with each other. In this case, the maps of two UAVs can be integrated into one map, which can be shared by the two UAVs in the following search process. The integration operator in this study is defined as ⊗, which can be described as Equation (7).

(7) $$S_{i}(g_{x,y},t)⊗S_{j}(g_{x,y},t)\Rightarrow \left\{\begin{array}{c} 1⊗1=1\\ -1⊗1=1\\ 0⊗0=0 \end{array}\right.$$

Obviously, the integration operator ⊗ satisfies the commutative law and associative law.

## 3. DE-Based Cooperative Search Strategy of Multiple UAVs

### 3.1. Planning Strategy Collaboration Using DMPC

Model predictive control (MPC), also known as rolling time domain control, is an optimal control method that divides the long-time span control problem into several short-time span control problems. When using UAVs to solve CPP problems, the path plan is a dynamic process. Concretely, each UAV in a swarm needs to optimize its search path at each time point *t* according to other UAVs’ conditions and the task completion of the CPP. Thus, in this study, DMPC is applied to the UAV-based path planning problem.

**Figure 4:**
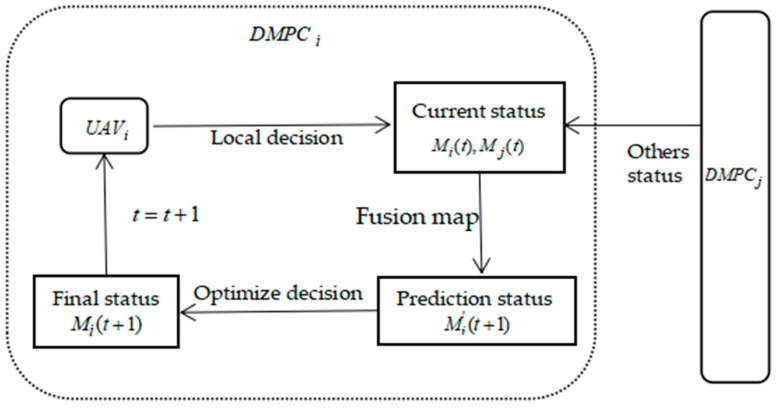
Decision-making process diagram of multi-UAV cooperative control based on DMPC.

The core concept of MPC is the division of an entire problem into current and future problems in a control cycle. Accordingly, the current problem can be solved or optimized based on the current system state, while some states of the future problems can be predicted. When using UAVs to solve a CPP problem, the coverage search problem is a dynamic optimization problem. Thus, we combine a distributed framework with MPC, named DMPC, to establish the UAV decision-making process, which is illustrated in Figure 4.

In Figure 4, DMPC*_i_* and DMPC_j_ (*j* ≠ *i*) represent the model predictive control system of *UAV_i_* and other UAVs, respectively. Figure 4 shows that, in the control cycle *t*, the decision-making process of *UAV_i_* is divided into three parts, as follows:

1. Current status acquisition. Each *UAV_i_* obtains its current local environment map *M_i_*(*t*) through local decision-making. Meanwhile, an environment map *M_j_*(*t*) of other *UAV_j_* in time *t* can also be obtained through a communication network.
2. System state prediction. Based on the current states *M_i_*(*t*) and *M_j_*(*t*), the predicted state *M’_j_*(*t* + 1) at time *t* can be obtained.
3. Decision optimization. The proposed DE is used to optimize the current decision, i.e., path planning, according to the prediction state and constraint quantity, and to obtain the final decision *M_i_*(*t* + 1) at time *t* + 1.

The system will update continuously with time according to the above decision-making process and obtain the final solution after all the time slices are used up.

**Figure 5:**
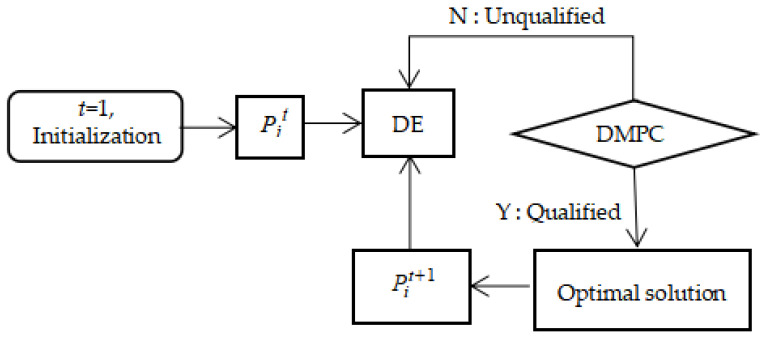
The flowchart of a UAV’s path planning framework.

In Figure 4, it can be seen that the DE-based decision optimization part plays a crucial role in improving the coverage search performance of multiple UAVs. Based on DMPC, the flowchart of a UAV’s path planning framework is shown in Figure 5, in which *P_i_^t^* denotes the best solution obtained (i.e., the best path) for *UAV_i_* at time *t*.

### 3.2. Path Generation Based on DE

DE is an outstanding heuristic optimization algorithm, and some favorable characteristics of DE, such as a low number of parameters, easy implementation, and parallelism, enable it to be successfully applied in many fields [28,29,30,31]. Thus, in this study, DE is adopted as an optimizer to plan UAVs’ coverage search paths. A standard DE consists of four distinct steps, i.e., initialization, mutation, crossover, and selection, the details of which are introduced as follows.

#### 3.2.1. Initialization

When utilizing DE to optimize a problem, the first issue is to obtain an initial population. Considering that a distributed path planning framework is adopted in this study, we design an improved DE based on multiple subpopulations. Then, each UAV evolves in a separate subpopulation *P_t_^i^* locally to find an optimized coverage search path.

In the initial stage of the search process, *NP* individuals ${\vec{X}}_{i,n}^{t}$ with *H* genes are randomly generated for each *UAV_i_*, where *t* represents the number of iterations and *H* denotes the steps in each plan path. In ${\vec{X}}_{i,n}^{t}$, each element represents the direction of each step; for example, ${\vec{X}}_{i,n}^{t}$ = {1, 1, 0, 0, −1} indicates that the turning directions of the next five successive steps are as follows: turn left, turn left, go straight, go straight, and turn right. The generalization formula of ${\vec{X}}_{i,n}^{t}$ in any number of iterations *t* be described as Equation (8).

(8) $${\vec{X}}_{i,n}^{t}=\left\{x_{i,n,1}^{t},\cdots x_{i,n,h}^{t},\cdots ,x_{i,n,H}^{t}\right\}\in P_{i}^{t}$$

where 1≤i≤NU, 1≤n≤NP, 1≤t≤T, and 1≤h≤H.

The initialization of the population is generated using a uniform random function. For example, the *h*-th position of an individual can be randomly initialized according to Equation (9).

(9) $$x_{i,n,h}^{1}=x_{\min}+rand\cdot \left(x_{\max}-x_{\min}\right)$$

where *x*_min_ and *x*_max_ represent the lower and upper boundaries, respectively, and *rand* is a random value in the interval [0, 1].

#### 3.2.2. Mutation

In each generation, each individual ${\vec{X}}_{i,n}^{t}$ undergoes a mutation operator to obtain a mutant vector ${\vec{V}}_{i,n}^{t}$. Generally, there are six widely applicable mutation operators. In this study, the basic mutation operator “DE/rand/1” is selected by each individual, and the operator is defined as Equation (10).

(10) $${\vec{V}}_{i,n}^{t}={\vec{X}}_{r1,n}^{t}+F\cdot \left({\vec{X}}_{r2,n}^{t}-{\vec{X}}_{r3,n}^{t}\right)$$

where ${\vec{X}}_{r1,n}^{t}$, ${\vec{X}}_{r2,n}^{t}$, and ${\vec{X}}_{r3,n}^{t}$ are three randomly selected individuals from a subpopulation that the individual ${\vec{X}}_{i,n}^{t}$ belongs to. F∈(0, 1) can be regarded as a scaling factor, and ${\vec{V}}_{i,n}^{t}=\left\{v_{i,n,1}^{t},v_{i,n,2}^{t},\cdots v_{i,n,H}^{t}\right\}$ is the generated mutant vector for the individual ${\vec{X}}_{i,n}^{t}$.

#### 3.2.3. Crossover

After the mutation operation, the original individual ${\vec{X}}_{i,n}^{t}$ and its mutant vector ${\vec{V}}_{i,n}^{t}$ need to be crossed to generate a new individual ${\vec{X}}_{i,n}^{t+1}=\left\{x_{i,n,1}^{t+1},x_{i,n,2}^{t+1},\cdots x_{i,n,H}^{t+1}\right\}$. Because there is no selection operation, the new individual will replace the old individual in the corresponding position in the original population directly. The formula of the crossover operation is described as Equation (11).

(11) $${x^{\prime }}_{i,n,h}^{t+1}=\left\{\begin{array}{l} v_{i,n,h}^{t},\;rand\leq CR\\ x_{i,n,h}^{t},\;other \end{array}\right.$$

where *CR* is a crossover rate, which determines whether $x_{i,n,h}^{t+1}$ is copied from $x_{i,n,h}^{t}$ or $v_{i,n,h}^{t}$.

Finally, each floating-point vector needs to be converted into an integer vector according to Equation (12).

(12) $${x^{\prime }}_{i,n,h}^{t+1}=\left\{\begin{array}{l} 1,{x^{\prime }}_{i,n,h}^{t}>\frac{1}{3}x_{\max}\\ 0,\;\mathrm{other}\;\\ -1,{x^{\prime }}_{i,n,h}^{t}<-\frac{1}{3}x_{\max} \end{array}\right.$$

where $x_{i,n,h}^{t+1}=\left\{-1,\;\;0,\;1\right\}$ indicates whether *UAV_i_* will turn left, right, or straight in the current direction in the *h*-th step.

#### 3.2.4. Selection

After obtaining the new path ${{\vec{X}}^{\prime }}_{i,n}^{t+1}$, its performance needs to be evaluated. Detailed information about the metrics and evaluation methods is provided in Section 4. Based on the results of the evaluation, the optimized path can be selected based on Equation (13)

(13) $${\vec{X}}_{i,n}^{t+1}=\left\{\begin{array}{l} {{\vec{X}}^{\prime }}_{i,n}^{t+1},\;\mathrm{if}\;fit({{\vec{X}}^{\prime }}_{i,n}^{t+1})\geq fit({\vec{X}}_{i,n}^{t})\\ {\vec{X}}_{i,n}^{t},\;\mathrm{else}\; \end{array}\right.$$

After *T* iterations of the above operators, i.e., mutation, crossover, and selection operators, we can obtain *NP* candidate coverage search paths. Then, the best path ${\vec{X}}_{i,best}^{t+1}$ measured by the evaluation metrics introduced in Section 4 is adopted as the search path of *UAV_i_* in the next search process.

## 4. Fitness of a Planning Path

When using DE to optimize search paths of multiple UAVs, the first issue is to determine how to evaluate a search path. In this study, a dynamic fitness function is proposed, in which energy consumption and coverage are two optimization objectives. The details of the fitness function are discussed as follows.

### 4.1. Coverage Rate

When performing a coverage search task, the coverage rate of a UAV is a core index that is used to evaluate its performance. The higher the coverage rate is, the greater the possibility that the task can be completed.

To compute the coverage rate of a planning path of *UAV_i_*, we need to determine the coverage of the two consecutive steps; the coverage increment of the UAV at the time *t* can be calculated according to Equation (14).

(14) $$\Delta Cov_{i}(t)=Cov_{i}(t)-Cov_{i}(t-1)$$

where the coverage rate $Cov_{i}(t)$ is calculated according to Equation (15).

(15) $$Cov_{i}(t)=\left|\sum_{x=1}^{Lx}\sum_{y=1}^{Ly}S_{i}(g_{x,y},t)\right|$$

Finally, the coverage evaluation function can be defined as Equation (16).

(16) $$R_{1}^{i}(t)=\frac{\Delta Cov_{i}(t)}{5(H-1)+{\left(2r+1\right)}^{2}}$$

where *H* is the number of search steps in each planning path, and *r* is the radius of a UAV’s view window. In this study, *H* and *r* are set as 7 and 1, respectively.

This equation is the normalization of the coverage increment intended to facilitate the calculation of the total reward function. The denominator of Equation (16) is the maximum coverage increment of the UAV fleet after *H* steps.

### 4.2. Energy Consumption Estimation

The energy consumption of a UAV is an important index for determining its performance. There are many factors that affect the energy consumption of a UAV, but there is no doubt that frequently changing direction always consumes more energy than a straight-line flight with a constant speed. Therefore, this study takes the total turning times of a UAV in each complete planning path as a criterion to evaluate the UAV’s energy consumption. The energy consumption evaluation function can be defined as Equation (17).

(17) $$R_{2}^{i}(t)=1-\frac{turnN(t)}{H}$$

where *turnN*(*t*) ∈ {1, 2, …, *H*} is the number of turns in the planning path at time *t* and *H* is the number of search steps in each planning path.

The maximum number of turns that a UAV completes in a single planning path is *H*. Thus, $R_{2}^{i}(t)$ epresents the ratio of the number of turns without turning to the step. The larger $R_{2}^{i}(t)$ indicates that the UAV has fewer turns in a single planning step and lower energy consumption.

### 4.3. Dynamic Fitness Function

In this study, we aim for multiple UAVs to perform efficient coverage searches in the search area. In practical applications, an island map may be non-convex and discrete. Thus, it is unavoidable that a UAV flies across the non-search areas. Therefore, in this study, it is also important to determine how to effectively deal with this situation and achieve better search results.

For example, if *UAV_i_* is located in a search area at time *t*, it should pay more attention to coverage as well as energy consumption. Conversely, if *UAV_i_* is located in a non-search area at time *t*, the priority of the UAV should be to find a route back to the search area rather than the coverage rate and energy consumption. In this case, the UAV needs to perform more turns to re-enter the search area. Thus, when a UAV is out of the search area, the evaluation metric can be defined as Equation (18).

(18) $$R_{3}^{i}(t)=\frac{turnN(t)}{H}$$

Based on the three evaluation metrics, which are separately defined as Equations (16)–(18), the fitness of *UAV_i_* can be evaluated based on Equation (19).

(19) $$fit({\vec{X}}_{i,n}^{t})=\left\{\begin{array}{l} \omega_{1}\cdot R_{1}^{i}(t)+\omega_{2}\cdot R_{2}^{i}(t),\;\mathrm{if}\;{\vec{X}}_{i,n}^{t}\;\notin M_{n}\\ \omega_{3}\cdot R_{1}^{i}(t)+\omega_{4}\cdot R_{3}^{i}(t),\;\mathrm{else}\; \end{array}\right.$$

where $\omega_{1}$~$\omega_{4}$ are four weights of the different evaluation metrics in different conditions.

The dynamic fitness function is used to evaluate the performance of each planning path of *UAV_i_*, and the path with the largest fitness value is the optimal solution for *UAV_i_*. Generally, the optimal solution of *UAV_i_* at time *t*, recorded as ${\vec{X}}_{i,best}^{t}$, is regarded as a real search path of *UAV_i_*.

### 4.4. Map Integration

To realize the cooperative coverage search of multiple UAVs, different UAVs need to share their search information through a communication process. Specifically, two different UAVs share and integrate their local maps and then update their map information according to Equation (7). Consequently, each UAV plans its own subsequent search path based on the updated map. Without loss of generality, this study defines the communication range of each UAV as a standard circle. When the Euclidean distance between two UAVs is in a preset communication range, the two UAVs can communicate with each other, and then, their map information can be integrated and shared.

### 4.5. Framework of DECSMU

Finally, based on the aforementioned discussions, the pseudocode of the proposed DECSMU is given in Algorithm 1.
**Algorithm 1.** DECSMU**Input:** Initialization: *t* = 1, $\omega_{1}$, $\omega_{2}$, $\omega_{3}$, $\omega_{4}$, ${\vec{X}}_{i,n}^{t}$ (1 ≤ *i* ≤ *NU*, 1 ≤ *n* ≤ *NP*);   *M_i_*, = *M* (1 ≤ *i* ≤ *NU*), where *M* is a rasterized map of the task area;1: **while** not satisfy stop conditions **do**2:   **for**
*i* = 1: *NU* **do** // *Nu* is the number of *UAVs*3:    **for** *j* = 1: *NP* **do** // *Np* is the number of paths for each UAV4:     Random generate ${\vec{X}}_{i,j}^{t}$;5:     Performing Mutation, Crossover, and Selection operators on ${\vec{X}}_{i,j}^{t}$ (see Section 3);6:     Generate ${\vec{X}}_{i,j}^{t+1}$;7:    **end for**8:    Obtain ${\vec{X}}_{i,best}^{t+1}$ from all the ${\vec{X}}_{i,j}^{t+1}$;9:   **end for**10:   Each UAV performing its search path according to the obtained ${\vec{X}}_{i,best}^{t+1}$;11:   **for** *i* = 1: *NU* **do**12:     **for** *j* = 1: *NU* **do**13:      Perform map cooperation based on *M_i_* and *M_j_* (see Section 4.4);14:     **end for**15:   **end for**16:   *t* = *t* + 1;17: **end while**18: Integrate local maps of all UAVs into a single map *M* according to Equation (7);19: **Return**
*M*.

Note that, in Algorithm 1, the mutation, crossover, and selection operators in line 5 are executed in multiple iterations rather than in a single iteration. In other words, the evolution process of DE is described by line 5 in Algorithm 1. Then, the optimal path (i.e., ${\vec{X}}_{i,j}^{t+1}$ in line 6) is obtained after the optimization process of DE.

## 5. Experiments and Discussion

### 5.1. Performance of Parameters

**Figure 6:**
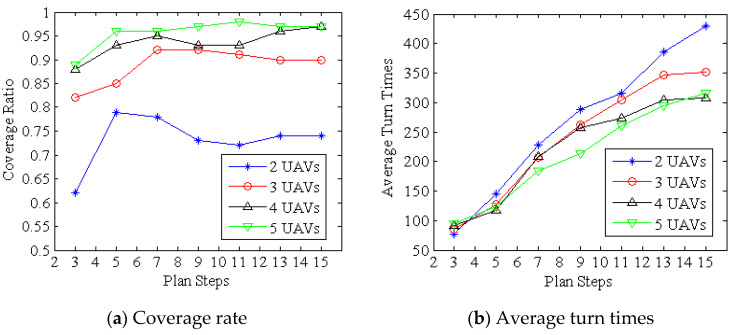
Performance of the number of UAVs and the plan steps in terms of the coverage rate and average turn time.

In DECSMU, many parameters determine its behavior. In this part, the performance of the two key parameters, i.e., the number of UAVs (*NU*) and the search step (*H*), are examined by a set of experiments. In the experiments, four different *NU* (*NU* = 2, 3, 4, and 5) and seven different *H* (*H* = 3, 5, 7, 9, 11, 13, and 15) are tested, and the experimental results are demonstrated in Figure 6. Note that all the experiments in this study are conducted based on the following systems:

- OS: Windows 10
- CPU: Intel i7-7700K, 4.20 GHz
- RAM: 16 GB
- Language: Matlab R2020a

In the experiments, two metrics are used to measure the performance of different values of the two parameters. One of the metrics is the coverage rate, which is the main objective of DECSMU. The other one is the average turn time of each UAV, which can be utilized to evaluate the energy consumption of the UAV.

In Figure 6a, we can see that DECSMU with two UAVs achieves the lowest coverage ratio, while DECSMU with five UAVs yields the highest coverage ratio. The results suggest that more UAVs can bring a higher coverage ratio. Meanwhile, it can also be seen that as the number of UAVs increases, the difference in the coverage ratio of DECSMU becomes smaller. For instance, the difference in the coverage ratio between four UAVs and five UAVs is less than 5% for the entire tested search step. Moreover, the experimental results also indicate that *H* = 5 or 7 can result in very favorable performance.

In addition, the experimental results illustrated in Figure 6b show that the average turn time of each UAV rises rapidly with an increase in plan steps. Moreover, we also notice that when using two UAVs, each one has a higher turn time in almost all the planned steps. In fact, the phenomenon does not mean that each UAV has a higher turn time than other conditions (such as three, four, and five UAVs). On the contrary, the phenomenon indicates that when using fewer UAVs to cooperate in the search process, each UAV needs to fly a longer distance.

According to the experimental results presented in Figure 6a,b, we can obtain the following preliminary conclusion. In this study, four and five UAVs can offer outstanding performance when *H* = 5 or 7. On the one hand, the configuration can bring a coverage ratio of more than 90% in the predefined search process. On the other hand, when utilizing four or five UAVs in DECSMU, each UAV has a lower turn time if *H* = 5 or 7. As a result, in the following experiments, four UAVs and *H* = 7 are adopted in DECSMU since we assume that the configuration can bring a balance between the coverage ratio and energy consumption.

### 5.2. Experimental Setup

To verify the performance of DECSMU, we set up a simulation environment for the coverage search of multiple UAVs and designed three experiments to verify the robustness of DECSMU in different search regions.

**Figure 7:**
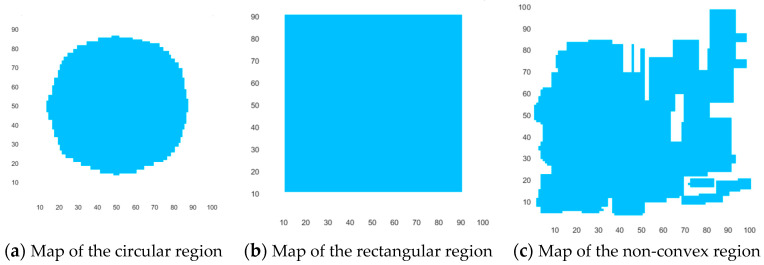
Maps of three different search regions in this study.

In the experiments, four UAVs, which are placed in different locations on the simulation task map, execute the coverage search. The size of the simulation task map is 100 × 100, the view radius of each UAV is *r* = 1, and the search step is *H* = 7, replacing time *t* with the number of iterations. The stop condition of DECSMU is that each UAV performs path planning 100 times. The three different search maps used in the experiments are illustrated in Figure 7, in which the blue part denotes the area that needs to be searched, while the white part represents the area that does not need to be searched. In this study, DE is utilized to optimize the coverage search path for each UAV. The parameters in DE are important for the coverage task. Thus, in this study, some popular parameters are set in advance, and a set of optimal parameters is adopted in the following study. These parameters include *NP* = 100, *T* = 100, *H* = 7, *F* = 0.5, and *CR* = 0.1. Four weights are set as follows: $\omega_{1}$ = 0.6, $\omega_{2}$ = 0.4, $\omega_{3}$ = 1.0, and $\omega_{4}$ = 0.

In this study, two convex regions (i.e., a circular region and a rectangular region) and a non-convex region are adopted to evaluate the performance of DECSMU. The maps of the three regions are illustrated in Figure 7.

### 5.3. Experimental Results and Discussion

In this section, the experimental results and discussion are presented. To verify the overall performance, DECSMU is executed through 30 independent runs on each map, and the results, in terms of the coverage ratio, are presented in Table 1.

In the table, we can see that DECSMU exhibits a very reliable performance for the different maps. Concretely, it achieves a coverage ratio of more than 90% for the three different maps, and each optimal search step can be obtained within 2 s. Moreover, it displays more outstanding convex maps, i.e., circular and rectangular maps. Although the results for the non-convex map are dominated by the results for the other two maps, DECSMU still offers acceptable performance for the complicated map.

**Figure 8:**
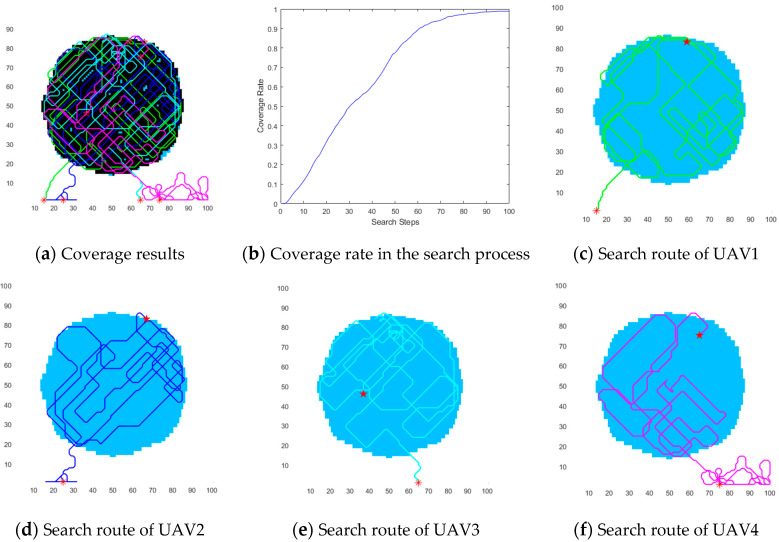
Coverage results within the circular region.

**Figure 9:**
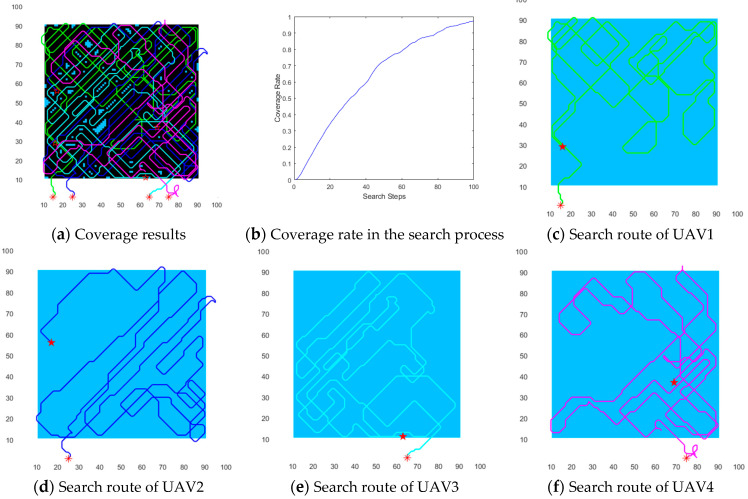
Coverage results within the rectangular region.

**Figure 10:**
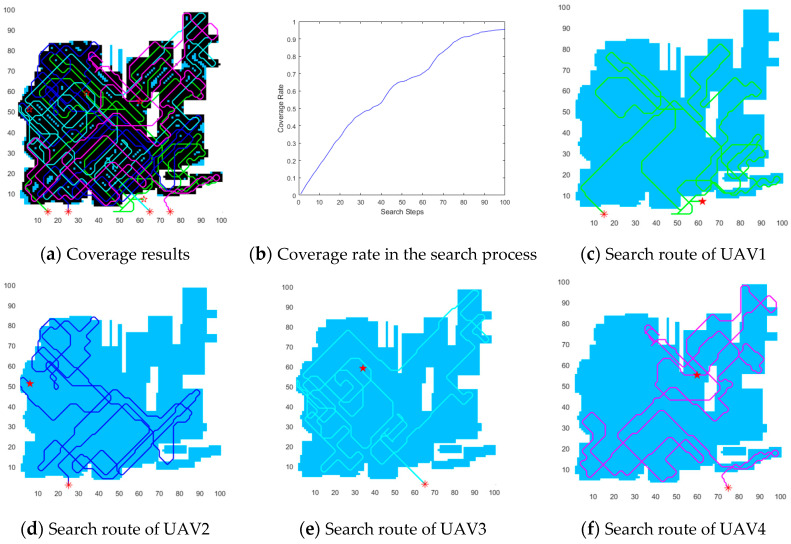
Coverage results within the non-convex region.

In order to describe the characteristics of DECSMU more intuitively, the coverage results for the three regions are demonstrated in Figure 8, Figure 9 and Figure 10, respectively. In the three figures, each symbol ‘*’ in the map represents the starting location of a UAV, and each symbol ‘★’ in the map represents the ending location of a UAV. Each UAV’s path is identified by a line of a different color, and the black area denotes the coverage area.

#### 5.3.1. Experimental Results for the Circular Region

Figure 8a shows that all four UAVs search within the circular region after they enter this region. Moreover, Figure 8b shows that the coverage rate of multiple UAVs is higher than 98% after 100 search steps. Figure 8b also shows that the coverage rate is more than 70% after 50 search steps. In Figure 8c,d we can see that all UAVs have similar energy consumption when they perform a coverage search within the circular region, except *UAV*_4_, which consumes more energy than the other three UAVs outside the circular region. However, after a few search steps, the UAV flies towards the circular region based on the dynamic reward function.

#### 5.3.2. Experimental Results for the Rectangular Region

The rectangular region is another convex area adopted in this experiment. From the result demonstrated by Figure 9a, we can observe that the cooperation of four UAVs can attain a very favorable coverage performance in the rectangular region. Concretely, in Figure 9b, one can see that the coverage rate of the four UAVs is more than 95% of the region after 100 search steps. Similar to the results for the circular region, it also can be observed from Figure 9b that the coverage rate is more than 70% after 50 search steps. Moreover, in Figure 9c–f, we can observe an interesting phenomenon, i.e., each UAV mainly searches within a region. For instance, *UAV*_1_ pays more attention to the upper region, and *UAV*_2_ focuses on the lower right corner of the rectangular region, while *UAV*_3_ focuses on the left corner of the region. The planned paths of all four UAVs indicate that multiple UAVs can cooperate effectively.

#### 5.3.3. Experimental Results for the Non-Convex Region

In the above experiments, two convex regions, i.e., the circular region and the rectangular region, were adopted. In fact, non-convex regions are more common in the real environment. Moreover, a non-convex region is more difficult to cover than a convex region. Thus, in this section, a non-convex region is selected to evaluate the performance of DECSMU, and the results of the experiments are demonstrated in Figure 10.

The experimental results demonstrated in Figure 10a show that the majority of the non-convex region was searched by the four UAVs, except for a few spikes on the region’s edge. Figure 10b indicates that the coverage rate of the four UAVs is about 95% of the difficult region after 100 search steps, which is slightly worse than the results for the rectangular region.

Furthermore, from the search paths of the four UAVs demonstrated in Figure 10c–f, we can observe that each UAV can rapidly re-fly into the target areas if the UAV has flown outside the target areas. The results show that the dynamic reward function is helpful for a UAV to plan efficient paths in different conditions.

## 6. Conclusions

In this paper, we proposed a multi-UAV-based cooperative coverage search algorithm to deal with the coverage problem in various regions. In the proposed algorithm, named DECSMU, each UAV independently uses DE to plan and optimize its search path. To reduce the complexity of the coverage problem, we introduced a DMPC strategy to establish the UAV decision-making process. Based on DMPC, the complexity of the coverage problem can be reduced effectively. Moreover, in order to solve some constraints in real environments, a dynamic reward function is proposed, in which the turning angle, communication constraints, energy consumption, and coverage are considered.

In order to evaluate the performance of the proposed method, two typical regions, i.e., a circular region and a rectangular region, and a non-convex region are adopted to verify the robustness, error correction ability, and energy saving ability of multiple UAVs. The experimental results demonstrated that DECSMU yields an outstanding and reliable performance in the three regions. Moreover, the favorable property of the dynamic reward function was also verified by a set of experiments. In future studies on DECSMU, we will improve its application in a more realistic communication environment, such as how to search more effectively when considering the communication tower.

## Figures and Tables

**Table 1:** The coverage results of DECSMU for the 3 maps.

	Results	Avg. (%)	Std.Dev. (%)	Best (%)	Time Usage (s)
Maps					
Circular		98.9	0.11	99.3	1.1
Rectangular		97.5	0.12	98.1	1.3
Non-Convex		94.4	0.15	95.6	1.4

## Data Availability

The data are contained within the article.
